# Plasma Ceramides Levels in Severe COVID-19 Disease: Correlations with Survival

**DOI:** 10.1093/ofid/ofag052

**Published:** 2026-02-05

**Authors:** Tri Pham, Andrea M Heredia Castillo, Andrew Atkinson, Linda R Peterson, M Cristina Vazquez Guillamet

**Affiliations:** Department of Medicine, Washington University in St.Louis School of Medicine, St. Louis, Missouri, USA; Division of Infectious Diseases, Department of Medicine, Washington University in St.Louis School of Medicine, St. Louis, Missouri, USA; Division of Infectious Diseases, Department of Medicine, Washington University in St.Louis School of Medicine, St. Louis, Missouri, USA; Institute for Informatics, Data Science, and Biostatistics, Washington University in St.Louis School of Medicine, St. Louis, Missouri, USA; Cardiovascular Division, Department of Internal Medicine, Washington University School of Medicine, St.Louis, Missouri, USA; Division of Infectious Diseases, Department of Medicine, Washington University in St.Louis School of Medicine, St. Louis, Missouri, USA; Division of Pulmonary and Critical Care, Department of Medicine, Washington University in St.Louis School of Medicine, St. Louis, Missouri, USA

**Keywords:** ceramides, coronavirus, COVID-19, lipidomics

## Abstract

**Background:**

Dysregulated inflammatory processes have been associated with severe COVID-19 disease. Ceramides, bioactive lipids involved in inflammatory signaling, have been utilized to predict outcomes in chronic cardiometabolic diseases and pancreatic cancer, but their role in COVID-19 remains unknown.

**Methods:**

This observational study prospectively enrolled patients with COVID-19 disease who required intubation and mechanical ventilation at a Midwestern academic hospital between August 2020 and March 2021. Plasma ceramides 16:0 (C16), 22:0 (C22), and 24:0 (C24) and inflammatory markers (CRP, ESR, D-Dimer, and ferritin) were collected and organized weekly from symptom onset for up to 6 weeks. Patients were grouped by in-hospital mortality status. Linear mixed-effects models assessed temporal trends and associations between ceramides, inflammatory markers, and clinical outcomes.

**Results:**

We enrolled 53 patients with COVID-19. Demographic and clinical characteristics were similar between survival groups, except nonsurvivors more frequently received convalescent plasma (77% vs 44%, *P* = .04). C16, C22, and C24 followed similar temporal trajectories to each other, but demonstrated an inverse pattern when compared with other inflammatory markers. Nonsurvivors demonstrated higher plasma levels of all the ceramide species in the first week of illness, followed by lower levels compared with survivors in subsequent weeks.

**Conclusions:**

The synchronous trajectories of C16, C22, and C24 differ from patterns reported in chronic disease and suggest their behavior may vary with disease acuity. Their inverse trend with standard inflammatory markers resembles the behavior of negative acute-phase reactants. Further research into ceramide flux dynamics in the acute-to-subacute phase of disease is needed.

## Teaser

Plasma ceramides levels in severe COVID-19 display distinct patterns compared with those observed in chronic cardiometabolic diseases, where ceramides are established prognostic indicators. Further investigation is needed to understand their potential clinical and prognostic role as inflammatory biomarkers.

Severe coronavirus disease 2019 (COVID-19) often manifests as acute respiratory distress syndrome (ARDS), leading to high mortality rates [1–3]. Understanding the mechanisms behind COVID-19 can inform development of prognostic biomarkers and future therapeutics. Thus far, viral-mediated tissue injury [4] from the severe acute respiratory syndrome coronavirus 2 (SARS-CoV-2) virus itself, and perhaps more importantly, dysregulated inflammatory responses [5, 6] have been implicated in severe presentations.

One area of interest involves understanding the inflammatory pathways underlying COVID-19. Ceramides, a type of bioactive sphingolipids, are mediators in cellular signaling and are often highlighted in inflammatory processes such as apoptosis and oxidative stress [7–9]. Ceramides have gained traction in cardiometabolic research, where compelling evidence suggests their role in accelerated atherosclerosis, insulin resistance, and cardiac tissue injury via modulated inflammatory signaling [10–14]. Elevated total plasma ceramide concentrations have been associated with worse outcomes, and reducing these levels have shown promise in improving prognosis [15]. Recent literature refines this understanding, indicating specific ceramide species hold distinct prognostic significance: increases in long-chain species (eg, ceramide 16:0 [C16]) and decreases in very-long chain species (eg, ceramide 22:0 [C22] and ceramide 24:0 [C24]) lead to worse outcomes [16, 17]. In other words, C16 is considered a “bad” ceramide, while C22 and C24 are considered the “good” ceramides. Importantly, a higher C24/C16 ratio has emerged as a robust predictor of more favorable prognoses [16].

Beyond their role in cardiovascular diseases, ceramides have also been implicated in the pathogenesis of numerous viral infections, facilitating viral entry and replication in pathogens such as human immunodeficiency virus-1, influenza A, measles, Ebola, and even SARS-CoV-2 [18–22]. More broadly, ceramides modulate host cellular responses in viral infections [18, 23] and may also contribute to the inflammatory cascade seen in severe COVID-19 disease. Supporting this, interleukin-6 (IL-6) [24] and C-reactive protein (CRP), 2 inflammatory markers predictive of COVID-19 outcomes [25–30], have been associated with elevated ceramide levels [13, 31]. Additionally, ceramides have also been implicated in pulmonary microvascular injury in COVID-19 disease [32, 33]. However, there is a dearth of information on the role of ceramide levels as prognostic markers in COVID-19.

Therefore, this study aimed to evaluate the association between ceramide levels and clinical outcomes in severe COVID-19 disease as well as examine the relationship between ceramides and other inflammatory markers throughout the disease course. We hypothesized that higher plasma levels of C16, along with lower levels of C22, C24, and C24/C16 ratio, would be associated with worse outcomes. We also hypothesized that C16 would positively correlate with traditional markers of inflammation, while C22 and C24 would inversely correlate.

## METHODS

### Study Design

This prospective observational cohort study was conducted at Washington University/Barnes-Jewish Hospital in St. Louis, MO between August 2020 and March 2021 (Institutional Review Board protocol: 202 006 151). Patients were eligible for enrollment upon admission to the medical intensive care unit if they were diagnosed with SARS-CoV-2 by nasopharyngeal polymerase chain reaction and required intubation and mechanical ventilation for ARDS, as defined by the Berlin criteria [34]. Informed consent was obtained from the legally authorized representative.

We collected demographic and clinical data, including: age, comorbidities, history of immunosuppressive therapies, treatments received for COVID-19, laboratory and inflammatory markers (including CRP, erythrocyte sedimentation rate [ESR], D-dimer, and ferritin), and clinical outcomes, such as in-hospital mortality, duration of intubation, and length of hospitalization. Day 0 was defined as the date of symptom onset (DSO). Plasma ceramides were collected at enrollment and targeted for at least weekly sampling throughout hospitalization for a maximum of 6 weeks. Conventional inflammatory markers were obtained as part of clinical care. While clinicians were encouraged weekly collection, they could order them more frequently at their discretion. Levels of ceramides were expressed in mg/mL, and prespecified ceramides of interest collected included C16, C22, and C24. Ceramides were collected and quantified in biobanked frozen plasma samples using liquid chromatography-tandem mass spectrometry assay as previously described [16, 35].

### Statistical Analyses

Organizing all data relative to DSO, we calculated mean values within each weekly bin when multiple measurements were available. We aimed to obtain 6 weekly values for each of the following biomarkers: C16, C22, C24, ESR, CRP, D-dimer, and ferritin. Patients were grouped by in-hospital mortality status (survivors vs nonsurvivors). Descriptive statistics were calculated for continuous variables, reported as the median and interquartile range (IQR), and categorical variables were expressed as frequencies and percentages. Group differences were assessed using the Mann–Whitney *U*-test for continuous variables and the χ² or Fisher's exact test (as appropriate) for categorical variables.

We examined the correlation between inflammatory markers and ceramide trajectories. To account for differences in reference ranges between ceramides and various inflammatory markers, we standardized all values using z-scores and set means to zero to facilitate comparison. Trends were assessed by fitting linear (mixed) models with the respective biomarker as dependent variable, both across the full 6-week period and cross-sectionally at weekly intervals.

In the primary analysis, we assessed the association between ceramide levels and in-hospital mortality. We generated box plots for each ceramide value, stratified by in-hospital mortality overall over the 6-week period. We fitted unadjusted linear mixed-effects models to identify trends in ceramide concentration between survivors and nonsurvivors, including a patient-level random intercept to account for the repeated measures. Next, univariable and multivariable mixed-effects regression models were fitted to identify factors associated with ceramide levels. We retained demographic and clinical variables with *P* values <.10 from univariable models in the adjusted models. This resulted in 4 final adjusted models, one for each ceramide species (C16, C22, and C24) along with the C24/C16 ratio. Prior to modeling, ceramide values were assessed for normality using histograms and Q-Q plots and log-transformed as needed to meet model assumptions. Missingness was present due to patients presenting to care after the first week of illness and occurrence of discharge/death before 6 weeks. We elected not to impute values given high biologic variability, nonrandom timing of draws and observational study design. All statistical analyses were performed using R version 4.4.2 (R Core Team. A language and environment for statistical computing. R Foundation for Statistical Computing [36–39]) between December 2024 and April 2025.

## RESULTS

We enrolled 53 patients with severe COVID-19 between August 2020 and March 2021. The median age was 65.4 years (IQR 59.7–73.8), and 27 individuals (50.9%) were male (Table 1). Eight patients (15.1%) had asthma, and 15 (28.3%) had chronic obstructive pulmonary disease (COPD). Eight participants (15.1%) were considered immunocompromised, with immunosuppressive therapies including lenalidomide–bortezomib–dexamethasone, sirolimus, mycophenolate, prednisone, tacrolimus, and azathioprine, all of which were initiated prior to DSO. Subjects were intubated at a median of 9 days (IQR 5–13) after DSO. Most patients received dexamethasone (98.1%), remdesivir (71.7%), and convalescent plasma (66%), while only 3 patients (5.7%) received tocilizumab. In-hospital mortality was 66%, and the median length of stay among survivors was 38.5 days (IQR 24.5–46), with the median time to death from DSO as 25 days (IQR 16.5–33). Overall demographic features and medical comorbidities did not significantly differ between survival groups. Treatment prevalence did not significantly differ between groups, except for convalescent plasma, which nonsurvivors received more frequently (77.1% vs 44.4%, *P* = .038). Survivors had more ventilator-free days compared with nonsurvivors (median 3 days [IQR 0–19.5] vs zero days [IQR 0–0], *P* < .001, Table 1).

**Table 1.** Demographic Data and Clinical Characteristics

Variables	Overall (n = 53)	Nonsurvivor (n = 35)	Survivor (n = 18)	*P* Value
Age, median (IQR), years	65.4 (59.7–73.8)	67.4 (61–74)	64 (53.8–70.9)	.079
Sex, n (%)				.440
Male	27 (50.9)	16 (45.7)	11 (61.1)	
Female	26 (49.1)	19 (54.3)	7 (38.9)	
Race, n (%)				.567
African American	22 (41.5)	16 (45.7)	6 (33.3)	
White	31 (58.5)	19 (54.3)	12 (66.7)	
Body mass index, median (IQR)	31.5 (27.7–43.4)	30.1 (25.9–41.5)	37 (30.7–46.1)	.086
Comorbidities, n (%)				
Asthma	8 (15.1)	3 (8.6)	5 (27.8)	.104
Cancer	2 (3.8)	2 (5.7)	0 (0)	.543
COPD	15 (28.3)	11 (31.4)	4 (22.2)	.702
Congestive heart failure	12 (22.6)	9 (25.7)	3 (16.7)	.730
Diabetes mellitus, type 2	31 (58.5)	21 (60.0)	10 (55.6)	.987
ESRD	6 (11.3)	4 (11.4)	2 (11.1)	.999
Immunosuppression	8 (15.1)	6 (17.1)	2 (11.1)	.701
Clinical characteristics and outcomes				
Max temperature, median (IQR), °C	38.9 (38.4–39.7)	38.8 (38.3–39.7)	39.2 (38.6–39.5)	.251
Days from DSO to max temperature, median (IQR)	11 (6–19)	12 (5–19.5)	10.5 (6.2–17.2)	.895
Max WBC, median (IQR), cells/μL	23.2 (19.7–29.5)	24.9 (20.2–29.2)	21.2 (19.2–33.4)	.599
Days from DSO to max WBC, median (IQR)	15 (10–20)	14 (10–19)	16.5 (11–24.8)	.535
Days from DSO to intubation, median (IQR)	9 (5–13)	9 (6.5–12)	6 (4–16.2)	.413
PaO2/FiO2 ratio at 24 h after intubation, median (IQR)	123 (89–208)	104 (81–176.5)	157 (112.8–226.8)	.028*
Deep vein thrombosis, n (%)	9 (17.0)	4 (11.4)	5 (27.8)	.245
Pulmonary embolism, n (%)	6 (11.3)	4 (11.4)	2 (11.1)	1.000
Dexamethasone, n (%)	52 (98.1)	34 (97.1)	18 (100.0)	1.000
Tocilizumab, n (%)	3 (5.7)	2 (5.7)	1 (5.6)	1.000
Convalescent Plasma, n (%)	35 (66.0)	27 (77.1)	8 (44.4)	.038*
Remdesivir, n (%)	38 (71.7)	25 (71.4)	13 (72.2)	1.000
Length of stay, median (IQR), days			38.5 (24.5–46)	
Ventilator-free days, median (IQR)	0 (0–0)	0 (0–0)	3 (0–19.5)	<.001*

This table summarizes the demographic features and aspects of the clinical course for the 53 enrolled participants. Data are presented for the overall cohort and then stratified by in-hospital survival status. Patients discharged from the hospital were classified as survivors, while those who died during the index hospitalization for COVID-19 were classified as nonsurvivors. Descriptive statistics were calculated for continuous variables, reported as the median and IQR, and categorical variables were expressed as frequencies and percentages. Group comparisons were conducted using the Mann–Whitney *U*-test for continuous variables, and the χ² or Fisher's exact test, as appropriate, for categorical variables.

*P value <.05.

Abbreviations: DSO, day of symptom onset; IQR, interquartile range; WBC, white blood cell.

### Temporal Trends in Ceramide Levels by Survival Status

**Figure 1.**
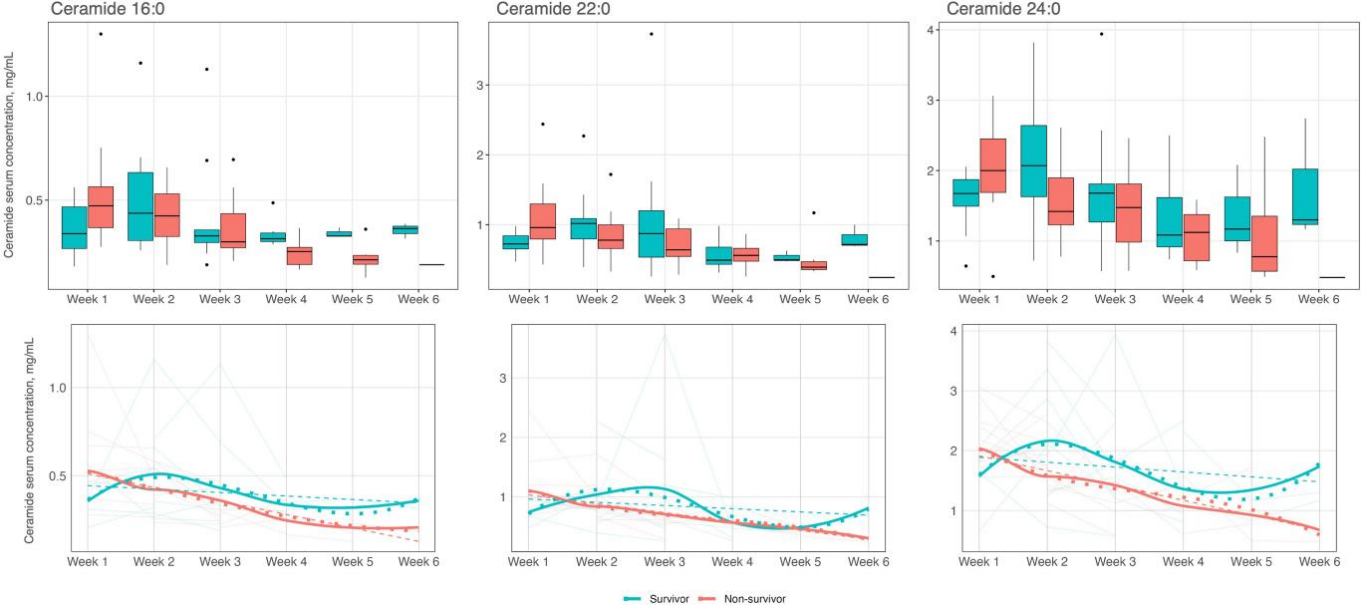
Plasma ceramide levels by survival status. This figure depicts ceramide levels by survival status. The first row presents the data as box plots, while the second row shows temporal trends using line graphs. In the second row, transparent solid lines indicate individual patient trajectories over time. The dashed line represents the overall linear trend. The dotted line shows a flexible spline-based trend using a cubic B-spline regression, and the solid bold line depicts a smoothed LOESS curve summarizing the average trend. Data presented in red represent nonsurvivors, while those presented in teal represent survivors. Abbreviations: LOESS, locally estimated scatterplot smoothing..

We evaluated ceramide levels by survival status using box plots and graphical depictions (Figure 1 and Supplementary Figure E1 in the supplement). Nonsurvivors had significantly lower C16 levels at week 4 compared with survivors (*P* = .033). They also had lower C24 and C16 levels at weeks 2 (*P* = .077) and 5 (*P* = .068), respectively, although these were not statistically significant. At week 1, C22 were higher in nonsurvivors compared with survivors, but this was also not statistically significant (*P* = .059). C24/C16 ratio did not exhibit statistically significant differences between mortality groups at any time point. A summary of ceramides and conventional inflammatory markers in the overall cohort and by survival status is provided in Table 2 and Supplementary Tables E1 and E2 in the supplement.

**Table 2.** Summary of Ceramide Species and Other Inflammatory Markers Across 6 Weeks

		Week 1	Week 2	Week 3	Week 4	Week 5	Week 6
Total Cohort	ESR, mm/h, median (IQR), n	54.5 (29–82), 5	78 (78–78), 1	71.5 (59.5–94.2), 4	95.2 (93.6–96.8), 2	79.5 (76.2–82.8), 2	100.5 (88.8–112.2), 2
	CRP, mg/mL, median (IQR), n	103.4 (78.4–149.7), 27	107.2 (74.6–151.8), 36	93.1 (37.5–161.7), 30	134.9 (88.7–173.4), 21	95.7 (59.3–184.3), 13	59.2 (51.8–69.7), 8
	D-Dimer, ng/mL median (IQR), n	1232.4 (924–3309.7), 27	3568.4 (1146.4–8041.3), 45	2346.2 (1293.1–4740.9), 32	3010.9 (1657.6–6900.2), 20	2078 (1724.4–9547.2), 14	2700.2 (1789.1–3353.8), 8
	Ferritin, µg/L, median (IQR), n	820.4 (364.1–1860.8), 26	810.4 (478.7–2225.5), 35	871.1 (577.6–1950.7), 27	1092 (777–2004.5), 17	1102.2 (487.4–2838.7), 12	720.6 (380.6–1444.4), 6
	C16, mg/mL, median (IQR), n	0.413 (0.304–0.504), 20	0.43 (0.317–0.547), 30	0.317 (0.272–0.426), 22	0.298 (0.264–0.335), 14	0.234 (0.208–0.327), 10	0.339 (0.284–0.369), 4
	C22, mg/mL, median (IQR), n	0.808 (0.709–0.982), 20	0.863 (0.67–1.056), 30	0.688 (0.54–0.945), 22	0.514 (0.464–0.666), 14	0.46 (0.372–0.502), 10	0.706 (0.582–0.787), 4
	C24, mg/mL, median (IQR), n	1.808 (1.619–2.244), 20	1.64 (1.25–2.144), 30	1.52 (1.148–1.81), 22	1.095 (0.845–1.418), 14	0.91 (0.678–1.582), 10	1.235 (0.998–1.66), 4
	C24/C16, mg/mL, median (IQR), n	4.3 (3.476–5.138), 20	3.737 (2.999–4.928), 30	5.008 (3.102–5.721), 22	3.577 (3.165–4.7), 14	4.107 (2.741–6.29), 10	3.648 (3.324–4.56), 4
Survivors	ESR, mm/h, median (IQR), n	68.2 (61.4–75.1), 2	NA	61 (58–71.5), 3	98.4 (98.4–98.4), 1	86 (86–86), 1	100.5 (88.8–112.2), 2
	CRP, mg/mL, median (IQR), n	135.4 (92–155.1), 11	89.2 (21.7–144.3), 14	56.8 (28.6–89.4), 14	105.4 (68.2–153.7), 10	96.8 (50–120), 6	64.8 (58.2–72.2), 6
	D-Dimer, ng/mL median (IQR), n	1158.2 (845.9–1621.5), 10	1348.8 (905.2–4223.8), 16	1843.2 (543.8–3952), 13	2155.8 (1090.2–2994.5), 8	2050.7 (1561.2–2720), 7	2529 (1765.5–3041.7), 5
	Ferritin, µg/L, median (IQR), n	500.5 (283.7–1857.2), 12	586.4 (278.4–2071.8), 14	645.5 (422.2–2141.4), 11	1769.3 (473.8–1985.7), 8	812.1 (398.7–3477), 6	720.6 (523.8–2000.5), 4
	C16, mg/mL, median (IQR), n	0.338 (0.267–0.468), 8	0.437 (0.306–0.633), 11	0.328 (0.296–0.356), 9	0.314 (0.3–0.342), 8	0.327 (0.326–0.348), 3	0.363 (0.339–0.374), 3
	C22, mg/mL, median (IQR), n	0.726 (0.654–0.842), 8	1.018 (0.796–1.089), 11	0.874 (0.537–1.2), 9	0.494 (0.434–0.679), 8	0.499 (0.489–0.564), 3	0.717 (0.706–0.858), 3
	C24, mg/mL, median (IQR), n	1.675 (1.494–1.872), 8	2.07 (1.63–2.64), 11	1.68 (1.27–1.81), 9	1.085 (0.916–1.617), 8	1.17 (1.002–1.625), 3	1.3 (1.235–2.02), 3
	C24/C16, mg/mL, median (IQR), n	4.698 (3.944–5.561), 8	4.416 (3.168–5.992), 11	5.122 (3.487–5.896), 9	3.193 (2.768–4.389), 8	3.589 (2.928–4.975), 3	3.714 (3.648–5.406), 3
Nonsurvivors	ESR, mm/h, median (IQR), n	29 (22.5–64), 3	78 (78–78), 1	131 (131–131), 1	92 (92–92), 1	73 (73–73), 1	NA
	CRP, mg/mL, median (IQR), n	92.2 (70.1–131.1), 16	112.2 (82.4–173.8), 22	153.9 (89.8–169.8), 16	147.7 (132.7–187.8), 11	95.7 (66.9–191.5), 7	34.1 (22.6–45.7), 2
	D-Dimer, ng/mL median (IQR), n	1768 (1014–5189.5), 17	4665.5 (1894–10 064.3), 29	3300 (1783.5–5250.8), 19	4262.6 (2044.7–13 213.8), 12	4448.5 (1726.2–27 554), 7	2871.5 (2334.2–3580.8), 3
	Ferritin, µg/L, median (IQR), n	1066.4 (697–1860.8), 14	925 (499.8–2052), 21	900 (758.8–1618.5), 16	893.7 (794.3–2686.3), 9	1367.1 (1063.2–2176.3), 6	892 (516–1268), 2
	C16, mg/mL, median (IQR), n	0.473 (0.368–0.565), 12	0.424 (0.325–0.53), 19	0.299 (0.27–0.435), 13	0.252 (0.19–0.272), 6	0.213 (0.192–0.234), 7	0.19 (0.19–0.19), 1
	C22, mg/mL, median (IQR), n	0.96 (0.795–1.297), 12	0.779 (0.66–0.998), 19	0.641 (0.548–0.94), 13	0.562 (0.48–0.662), 6	0.394 (0.358–0.472), 7	0.246 (0.246–0.246), 1
	C24, mg/mL, median (IQR), n	2 (1.69–2.45), 12	1.42 (1.23–1.898), 19	1.475 (0.984–1.81), 13	1.122 (0.72–1.376), 6	0.778 (0.572–1.352), 7	0.484 (0.484–0.484), 1
	C24/C16, mg/mL, median (IQR), n	4.131 (3.475–4.929), 12	3.289 (3.005–4.345), 19	5 (2.974–5.453), 13	3.947 (3.767–4.698), 6	4.624 (2.773–6.484), 7	2.551 (2.551–2.551), 1

This table summarizes the values of 4 commonly used inflammatory markers in the clinical setting alongside 3 ceramide species of interest. All values were averaged and organized on a weekly basis using the DSO as the point of reference, with day 0 marking the onset. Given varying clinical trajectories (such as death or discharge prior to week 6) and differences in the timing of hospital presentation, there are varying numbers of collected samples across the 6-week period. The first third of the table represents the entire cohort (n = 53), the middle third represents the survivors (n = 18), and the bottom third represents the nonsurvivors (n = 35).

Abbreviations: C16, ceramide 16:0; C22, ceramide 22:0; C24, ceramide 24:0; CRP, C-reactive protein; DSO, day of symptom onset; ESR, erythrocyte sedimentation rate; IQR, interquartile range.

To evaluate ceramide levels over 6 weeks, we fitted linear mixed models to assess differences between survival groups, fitting models to each of the ceramide biomarkers in turn (Table 3). Nonsurvivors had steeper decline in C16 levels relative to survivors in week 2 (β = −.390, *P* = .049), week 5 (β = −.730, *P* = .017), and week 6 (β = −.929, *P* = .041). Nonsurvivors had steeper decline in C22 (β = −1.241, *P* = .024) and in C24 (β = −1.241, *P* = .024) both at week 6. Lastly, when evaluating the C24/C16 ratio, no consistent trends were observed across the weeks.

**Table 3.** Estimates From the Fitted Linear Mixed-effects Models for Ceramide Species of Interest

Variables	C16			C22			C24			C24/C16		
	Estimate	SE	*P* Value	Estimate	SE	*P* Value	Estimate	SE	*P* Value	Estimate	SE	*P* Value
Week 1	REF	–	–	REF	–	–	REF	–	–	REF	–	–
Week 2	0.234	0.150	.124	0.168	0.187	.373	0.244	0.199	.224	−0.032	0.160	.843
Week 3	0.026	0.160	.872	0.058	0.199	.770	0.030	0.210	.886	−0.047	0.171	.784
Week 4	−0.094	0.171	.583	−0.426	0.210	.046*	−0.207	0.220	.351	−0.130	0.183	.479
Week 5	−0.019	0.246	.939	−0.446	0.295	.134	−0.202	0.305	.508	−0.251	0.266	.347
Week 6	0.016	0.243	.948	−0.017	0.294	.953	0.065	0.304	.832	0.035	0.262	.894
Survival status: nonsurvivor^a^	0.286	0.168	.092	0.170	0.200	.399	0.179	0.206	.386	−0.170	0.183	.355
Week 2 × nonsurvivor^b^	−0.390	0.194	.049*	−0.363	0.240	.136	−0.471	0.255	.070	−0.026	0.207	.900
Week 3 × nonsurvivor^b^	−0.378	0.214	.082	−0.424	0.263	.111	−0.371	0.276	.182	0.079	0.230	.733
Week 4 × nonsurvivor^b^	−0.438	0.249	.082	−0.104	0.302	.731	−0.387	0.315	.222	0.093	0.268	.730
Week 5 × nonsurvivor^b^	−0.730	0.299	.017*	−0.260	0.360	.473	−0.502	0.372	.180	0.301	0.324	.354
Week 6 × nonsurvivor^b^	−0.929	0.446	.041*	−1.241	0.540	.024*	−1.304	0.558	.022*	−0.185	0.481	.701

This table summarizes the estimates of 4 separate linear mixed-effects model, 1 for each ceramide species of interest along with 1 for the C24/C16 ratio. The models included survival status and its interaction with week (relative to DSO) to assess how these factors affected levels of each ceramide species. Across 53 patients, there were 100 observations for each ceramide species: C16, C22, and C24.

^a^The reference group for survival status is survivors.

^b^The reference group is week 1 × survivor.

*P value <.05.

Abbreviations: C16, ceramide 16:0; C22, ceramide 22:0; C24, ceramide 24:0; DSO, day of symptom onset; SE, standard error.

To adjust for potential confounders in these models, multivariable analyses were conducted, including demographic and clinical variables along with survival status (Supplementary Table E3 in the supplement). For C16, both sex and receipt of convalescent plasma were significant in the unadjusted model, but only convalescent plasma remained significant at the *P* < .05 level after adjustment. For C22, age, BMI, asthma history, and time from DSO to intubation were significant in the unadjusted model, but none remained significant after adjustment. For C24, age, BMI, and *P*/*F* ratio were significant in unadjusted analyses, but none remained significant after adjustment. Finally, for C24/C16 ratio, cancer, end-stage renal disease (ESRD), and receipt of convalescent plasma were significant in unadjusted analyses; cancer and ESRD remained significant in the adjusted model.

### Association Between Ceramide Levels and Inflammatory Markers

**Figure 2.**
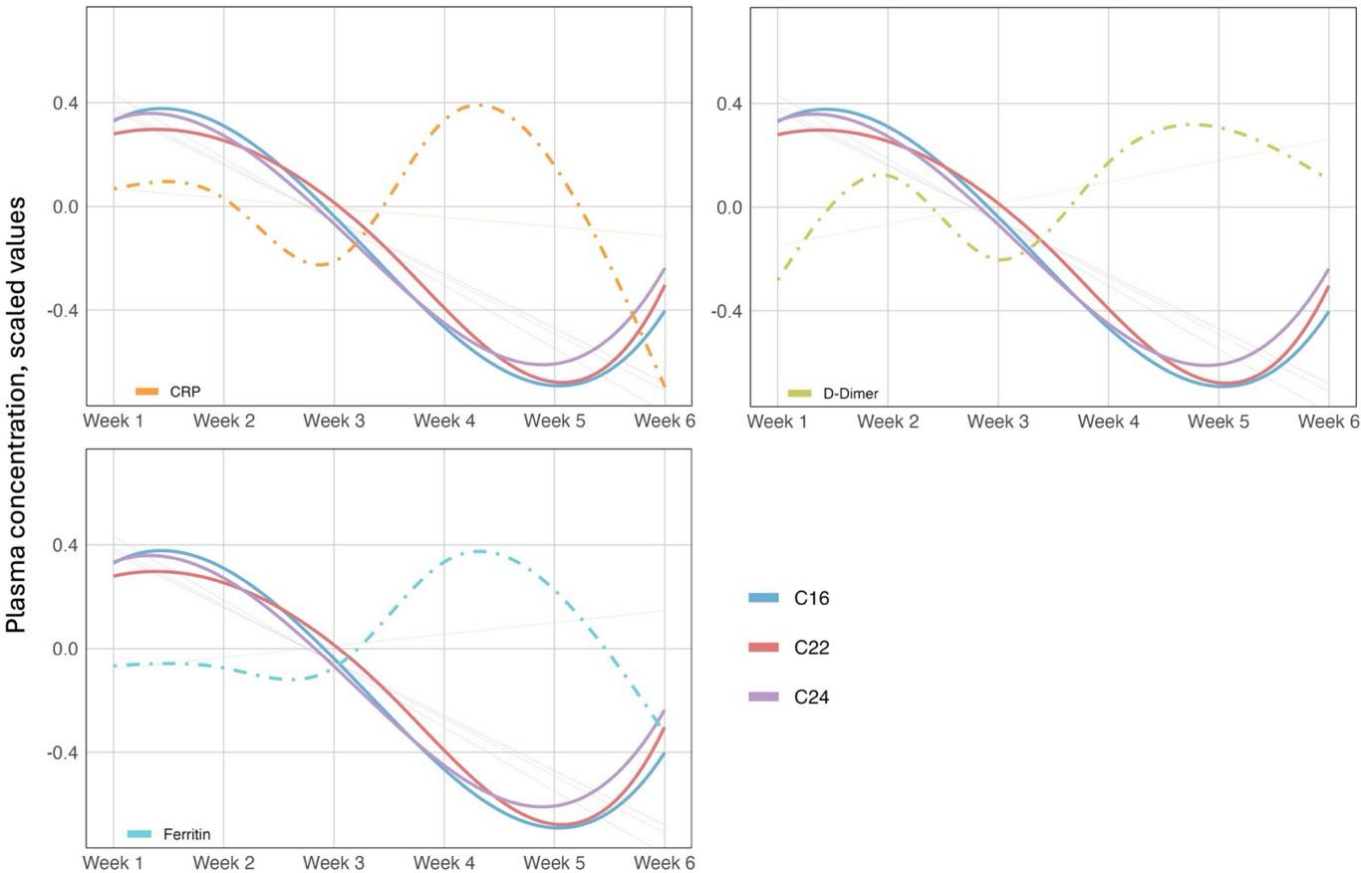
Scaled values of ceramide species and inflammatory markers. This figure represents the 3 ceramide species of interest (C16, C22, and C24) graphed with the more commonly used inflammatory markers. The first row displays results of CRP and D-Dimer, while the bottom row displays ferritin. ESR was excluded due to limited data points to reliably estimate a trend. To account for differences in reference ranges across biomarkers, we standardized all values using z-scores and set means to zero to facilitate comparison. The transparent dotted lines represent the overall linear trend. The solid curved lines represent the flexible spline-based trend for each ceramide species using a cubic B-spline regression, while the dotted-dashed line represents the flexible spline-based trend for each inflammatory marker. Abbreviations: C16, ceramide 16:0; C22, ceramide 22:0; C24, ceramide 24:0; CRP, C-reactive protein; ESR, erythrocyte sedimentation rate.

We plotted scaled values of ceramide species with inflammatory markers. While ceramides shared the same trajectory, an *inverse relationship* could be noted when comparing them to the other markers: increases in ceramide levels were associated with lower levels of inflammatory markers, while reductions in ceramide levels correlated with elevated inflammatory markers (Figure 2).

## DISCUSSION

This is the first study into the utility of specific plasma ceramide concentrations as predictors of mortality in patients with severe COVID-19. Contrary to our hypothesis that C16 would show associations with outcomes opposite to those of C22 and C24, there was synchrony across all measured ceramides. Nonsurvivors demonstrated higher plasma levels of C16, C22, and C24 in the first week of illness, followed by lower levels compared with survivors in all subsequent weeks. Moreover, there was generally an inverse relationship noted between ceramides and the inflammatory markers of interest in our study. These findings may offer preliminary insights into the role of ceramides in severe COVID-19, their metabolic flux, and their relationship with biomarkers of inflammation such as ESR and CRP.

Most research exploring ceramides has been in the context of chronic cardiometabolic illnesses, where ceramides have been shown as independent predictors of adverse cardiovascular events even in models with low-density lipoprotein cholesterol [40]. Beyond cardiovascular disease, ceramides also contribute to pulmonary vascular dysfunction [41] and, as such, have been implicated in pulmonary disorders. Notably, even across different disease spectrums, C16 is generally regarded as proinflammatory (ie, “bad” ceramide) while C22 and C24 are generally viewed as protective (ie, “good” ceramides) [16, 17].

Our findings partially aligned with the broader literature. As expected, nonsurvivors generally had higher levels of conventional inflammatory biomarkers (Supplementary Table E2 in the supplement), consistent with existing data [42–47]. In contrast, ceramides diverged from our hypothesis. While nonsurvivors had lower levels of the “good” ceramides (C22 and C24), they also had lower levels of the “bad” (C16). The low plasma C16 levels in both groups was unanticipated as historical observations in both COVID-19 and non-COVID-19 contexts have generally noted opposite associations with all-cause mortality between C16 with C22 and C24 [16, 17, 32, 33, 48].

One explanation could relate to disease acuity. Akin to cholesterol biomarkers, ceramides can predict mortality in patients with *stable* coronary heart disease [49], and as such, their plasma levels are typically obtained in ambulatory clinic patients [16, 50]. In contrast, our study collected ceramide in critically ill individuals. This acute-versus-chronic distinction is supported in the literature. In patients with ST-segment elevation myocardial infarction, both C16 and C24 significantly increase prior to reperfusion [15], demonstrating congruent dynamics in the acute setting but opposing effects in the chronic disease equivalence. Ceramides also increase with other acute disease states, such as sepsis [51] and subarachnoid hemorrhage [52]. In COVID-19, specifically, mechanisms leading to ceramide accumulation are acutely enhanced (eg, increased de novo synthesis [53] and upregulated sphingomyelinase activity [53, 54], and decreased ceramidase activity [55]). This may explain why, despite exhibiting distinct serologic roles in chronic diseases, all 3 ceramides concordantly existed in higher concentrations in the first week of illness in nonsurvivors compared with survivors in our study.

After the first week, all 3 ceramides declined more in nonsurvivors compared with survivors. We hypothesize this could reflect alterations in hepatic metabolism of lipoproteins during prolonged illness, analogous to suppression of liver-derived molecules commonly seen in systemic inflammatory states [56]. In fact, the liver is thought to be a major source of plasma ceramides [57]. Given that hepatic dysfunction develops in nearly half of hospitalized COVID-19 patients [58], it is possible that transcriptional shifts [59] can shunt pathways away from those that promote ceramide accumulation in postacute phase. However, studies documenting serial ceramide levels in the subacute phase of disease are sparse, so kinetics of ceramides over this period remains largely unknown. Another consideration is the difference in treatment exposure: a higher proportion of nonsurvivors received convalescent plasma, which may lead to alterations in sphingolipid pathways [60]. This would be supported by our results showing convalescent plasma was significantly associated with C16 levels in both unadjusted and adjusted models. Further, COVID-19 may become progressively less influential on ceramide metabolism, especially as complications from prolonged hospitalization (eg, hospital-acquired infections, critical illness myopathy, catabolism, pressure injuries, etc.) increasingly emerge with time. Early mortality and hospital discharge in nonsurvivors and survivors, respectively, may also limit the captured impact of COVID-19 on later ceramide trends.

Compared with trajectories of conventional inflammatory markers, ceramides generally displayed an inverse relationship over time. This was expected in long-chain ceramides such as C22 and C24 but unexpected in C16; however, such associations are derived from chronic disease contexts, as previously noted [50]. The overall ceramide trajectory in our cohort exhibits a behavior reminiscent to that of negative acute-phase reactants such as albumin or transferrin, both of which are suppressed in COVID-19 infections [61, 62]. While it is premature to concretely link acute ceramide concentrations with COVID-19 outcomes, this does suggest a correlation with established indicators of prognosis. More research is needed to elucidate the acute temporal dynamics of ceramides and the role, if any, they play alongside existing conventional inflammatory biomarkers to potentially provide a more nuanced sense of risk stratification in COVID-19.

This study has several limitations. First, this was conducted at a single center with 53 patients. While a small number in absolute terms, it is large compared with other severe COVID-19 biomarker studies [32, 33]. Second, our cohort is comprised of a homogenous population of critically ill patients with a high mortality rate, which could limit comparisons to prior studies that included a broader spectrum of illness severity and healthy controls. Third, ceramide and inflammatory biomarker data were not universally available across the study period. Varying clinical trajectories led to death or discharge at different time points prior to week 6, and not all patients presented to the hospital in the first week of disease onset, limiting opportunities for plasma collection. This longitudinal missing data, characteristic of many COVID-19 longitudinal studies, reduced our study power and limited additional analyses such as fitting of time-to-event models. Fourth, confounding factors, including unmeasured variables or unaccounted treatment effects, may have also influenced our results.

Our first-of-its-kind study offers preliminary insights into the association between plasma ceramides with mortality in COVID-19 patients. Our results were contrary to our initial hypothesis, suggesting unexplored pathophysiologic mechanisms in the acute COVID-19 that warrants further investigation. It is tempting to speculate that liver function differences relating to ceramide production underlies some of the differences seen in plasma ceramide levels and outcomes. At present, there is insufficient data to position ceramides above conventional inflammatory markers (or even to support their routine clinical use in COVID-19), given absence of widespread assay availability, standardized reference ranges, and evidence identifying which species and time points are most informative for patient care. Looking ahead, ceramides may also help elucidate one unique aspect of COVID-19 disease beyond risk stratification: inflammatory phenotypes. Emerging evidence supports the existence of hypoinflammatory and hyperinflammatory subtypes with differential responses to therapeutics [63–66]. Current approaches to define these phenotypes rely on cytokine levels and viral load. Future research can explore if ceramides can meaningfully contribute to these approaches [53].

In summary, plasma concentrations of C16, C22, and C24 were higher in the first week of severe COVID-19 in nonsurvivors compared with survivors, with levels subsequently declining more sharply in nonsurvivors. The congruence between ceramide species is unanticipated based on prior literature on ceramide metabolism in chronic disease, suggesting their behavior may differ based on disease acuity. Interestingly, plasma levels of these ceramides were inversely proportional to plasma levels of common inflammatory markers. While these findings provide preliminary insights into ceramide dynamics in COVID-19, further research is needed to better understand their trajectory in the acute-to-subacute phase of disease.

## Supplementary Material

ofag052_Supplementary_Data

## Supplementary Data

Supplementary materials are available at *Open Forum Infectious Diseases* online. Consisting of data provided by the authors to benefit the reader, the posted materials are not copyedited and are the sole responsibility of the authors, so questions or comments should be addressed to the corresponding author.
