# Supplementary material for: Plasma Ceramides Levels in Severe COVID-19 Disease: Correlations with Survival
Source: Open Forum Infect Dis. 2026 Feb 5;13(2):ofag052. doi: 10.1093/ofid/ofag052 (PMC12911038; doi:10.1093/ofid/ofag052)
Supplement: ofag052_Supplementary_Data [file ofag052_supplementary_data.docx]

**SUPPLEMENT FIGURES AND TABLES**

**Table E1. Comparison of differences between levels of ceramide species between survival status across six weeks.**

| **Variable** | **Survivors median (IQR), mg/mL** | **Non-Survivors median (IQR), mg/mL,** | **Survivors, n** | **Non-Survivors, n** | **p-value** |
| --- | --- | --- | --- | --- | --- |
| Week 1: C16 | 0.339 (0.267–0.468) | 0.473 (0.367–0.565) | 8 | 12 | 0.132 |
| Week 1: C22 | 0.726 (0.654–0.842) | 0.960 (0.795–1.297) | 8 | 12 | 0.059 |
| Week 1: C24 | 1.675 (1.494–1.873) | 2.000 (1.690–2.450) | 8 | 12 | 0.153 |
| Week 1: C24/C16 | 4.698 (3.944–5.561) | 4.131 (3.475–4.929) | 8 | 12 | 0.335 |
| Week 2: C16 | 0.437 (0.305–0.633) | 0.424 (0.325–0.530) | 11 | 19 | 0.547 |
| Week 2: C22 | 1.018 (0.796–1.089) | 0.779 (0.660–0.998) | 11 | 19 | 0.212 |
| Week 2: C24 | 2.070 (1.630–2.640) | 1.420 (1.230–1.897) | 11 | 19 | 0.077 |
| Week 2: C24/C16 | 4.416 (3.168–5.992) | 3.289 (3.005–4.345) | 11 | 19 | 0.322 |
| Week 3: C16 | 0.328 (0.296–0.356) | 0.299 (0.271–0.435) | 9 | 13 | 0.713 |
| Week 3: C22 | 0.874 (0.537–1.200) | 0.641 (0.548–0.940) | 9 | 13 | 0.64 |
| Week 3: C24 | 1.680 (1.270–1.810) | 1.475 (0.984–1.810) | 9 | 13 | 0.367 |
| Week 3: C24/C16 | 5.122 (3.487–5.896) | 5 (2.974–5.453) | 9 | 13 | 0.463 |
| Week 4: C16 | 0.314 (0.300–0.342) | 0.253 (0.191–0.273) | 8 | 6 | **0.033*** |
| Week 4: C22 | 0.493 (0.434–0.679) | 0.562 (0.480–0.662) | 8 | 6 | 0.846 |
| Week 4: C24 | 1.085 (0.916–1.617) | 1.123 (0.720–1.376) | 8 | 6 | 0.747 |
| Week 4: C24/C16 | 3.193 (2.768–4.389) | 3.947 (3.767–4.698) | 8 | 6 | 0.138 |
| Week 5: C16 | 0.327 (0.327–0.348) | 0.213 (0.192–0.234) | 3 | 7 | 0.068 |
| Week 5: C22 | 0.499 (0.489–0.564) | 0.394 (0.358–0.472) | 3 | 7 | 0.254 |
| Week 5: C24 | 1.170 (1.002–1.625) | 0.778 (0.572–1.353) | 3 | 7 | 0.362 |
| Week 5: C24/C16 | 3.589 (2.928–4.975) | 4.624 (2.773–6.484) | 3 | 7 | 0.82 |
| Week 6: C16 | 0.363 (0.339–0.374) | 0.190 (0.190–0.190) | 3 | 1 | 0.371 |
| Week 6: C22 | 0.717 (0.706–0.858) | 0.246 (0.246–0.246) | 3 | 1 | 0.371 |
| Week 6: C24 | 1.300 (1.235–2.020) | 0.483 (0.483–0.483) | 3 | 1 | 0.371 |
| Week 6: C24/C16 | 3.714 (3.648–5.406) | 2.551 (2.551–2.551) | 3 | 1 | 0.371 |

This table displays the median and IQR for each ceramide species of interest in our study by each week, setting DSO as the reference of day 0. Mann-Whitney U tests were conducted across all weeks. Bolded numbers with an asterisk represent p-value <0.05.

*Abbreviations used*: C16, ceramide 16:0; C22, ceramide 22:0; C24, ceramide 24:0; DSO, date of symptom onset; IQR, interquartile range

**Table E2. Comparison of differences between levels of conventional inflammatory markers between survival status across six weeks.**

| **Variable** | **Survivors median (IQR), mg/mL** | **Non-Survivors median (IQR), mg/mL,** | **Survivors, n** | **Non-Survivors, n** | **p-value** |
| --- | --- | --- | --- | --- | --- |
| Week 1: ESR | 68.25 (61.375-75.125) | 29 (22.5-64) | 2 | 3 | 0.773 |
| Week 1: CRP | 135.4 (91.95-155.15) | 92.15 (70.125-131.15) | 11 | 16 | 0.505 |
| Week 1: D-Dimer | 1,158.2 (845.875-1,621.5) | 1768 (1,014-5,189.5) | 10 | 17 | 0.219 |
| Week 1: Ferritin | 500.5 (283.7-1,857.25) | 1,066.4 (697-1,860.75) | 12 | 14 | 0.227 |
| Week 2: ESR | -- | 78 (78-78) | 0 | 1 | -- |
| Week 2: CRP | 89.15 (21.675-144.3) | 112.2 (82.45-173.775) | 14 | 22 | 0.072 |
| Week 2: D-Dimer | 1,348.8 (905.25-4,223.8) | 4,665.5 (1,894-10,064.3) | 16 | 29 | **0.022*** |
| Week 2: Ferritin | 586.35 (278.35-2,071.75) | 925 (499.8-2,052) | 14 | 21 | 0.219 |
| Week 3: ESR | 61 (58-71.5) | 131 (131-131) | 3 | 1 | 0.371 |
| Week 3: CRP | 56.75 (28.6-89.425) | 153.95 (89.8-169.8) | 14 | 16 | **0.044*** |
| Week 3: D-Dimer | 1,843.2 (543.8-3,952) | 3,300 (1,783.5-5,250.8) | 13 | 19 | 0.071 |
| Week 3: Ferritin | 645.5 (422.25-2,141.4) | 900 (758.85-1,618.475) | 11 | 16 | 0.474 |
| Week 4: ESR | 98.4 (98.4-98.4) | 92 (92-92) | 1 | 1 | 1.000 |
| Week 4: CRP | 105.4 (68.2-153.675) | 147.7 (132.7-187.8) | 10 | 11 | 0.113 |
| Week 4: D-Dimer | 2,155.75 (1,090.25-2,994.525) | 4,262.55 (2,044.7-13,213.8) | 8 | 12 | 0.177 |
| Week 4: Ferritin | 1,769.35 (473.775-1,985.675) | 893.7 (794.3-2,686.3) | 8 | 9 | 0.810 |
| Week 5: ESR | 86 (86-86) | 73 (73-73) | 1 | 1 | 1.000 |
| Week 5: CRP | 96.761 (49.962-120.046) | 95.7 (66.879-191.537) | 6 | 7 | 0.617 |
| Week 5: D-Dimer | 2,050.667 (1,561.25-2,720.024) | 4,448.5 (1,726.25-27,554.025) | 7 | 7 | 0.371 |
| Week 5: Ferritin | 812.071 (398.679-3,477) | 1,367.125 (1,063.25-2,176.312) | 6 | 6 | 0.689 |
| Week 6: ESR | 100.5 (88.75-112.25) | -- | 2 | 0 | -- |
| Week 6: CRP | 64.80 (58.18-72.24) | 34.13 (22.56-45.69) | 6 | 2 | 0.243 |
| Week 6: D-Dimer | 2,529 (1765.5-3,041.714) | 2,871.5 (2,334.25-3,580.75) | 5 | 3 | 0.766 |
| Week 6: Ferritin | 720.575 (523.8-2,000.455) | 892 (516-1,268) | 4 | 2 | 0.817 |

This table displays the median and IQR for the conventional inflammatory markers in our study by each week, setting DSO as the reference of day 0. Mann-Whitney U tests were conducted across all weeks. Bolded numbers with an asterisk represent p-value <0.05.

*Abbreviations used*: CRP, C-reactive protein; DSO, day of symptom onset; ESR, erythrocyte sedimentation rate; IQR, interquartile range

**Table E3. Estimates from the adjusted linear mixed-effects models for ceramide species of interest.**

| **Variables** | **Univariable Models** | | | | | | | | | | | | **Multivariable Models** | | | | | | | | | | | | | |
| --- | --- | --- | --- | --- | --- | --- | --- | --- | --- | --- | --- | --- | --- | --- | --- | --- | --- | --- | --- | --- | --- | --- | --- | --- | --- | --- |
|  | **C16** | | | **C22** | | | **C24** | | | **C24/C16** | | | **C16** | | | **C22** | | | **C24** | | | | **C24/C16** | | | |
|  | **Estimate** | **SE** | **P-Value** | **Estimate** | **SE** | **P-Value** | **Estimate** | **SE** | **P-Value** | **Estimate** | **SE** | **P-Value** | **Estimate** | **SE** | **P-Value** | **Estimate** | **SE** | **P-Value** | **Estimate** | **SE** | **P-Value** | **Estimate** | | **SE** | **P-Value** |  |
| Age | -0.008 | 0.004 | 0.072 | -0.012 | 0.005 | **0.010*** | -0.012 | 0.004 | **0.009*** | -0.004 | 0.004 | 0.297 | 0.000 | 0.004 | 0.943 | -0.005 | 0.005 | 0.332 | -0.004 | 0.005 | 0.458 |  | |  |  |  |
| Male sex (Ref: Female) | -0.215 | 0.103 | **0.041*** | -0.164 | 0.116 | 0.165 | -0.087 | 0.117 | 0.461 | 0.124 | 0.099 | 0.216 | -0.154 | 0.092 | 0.103 |  |  |  |  |  |  |  | |  |  |  |
| White race (Ref: Black race) | -0.036 | 0.107 | 0.738 | -0.102 | 0.118 | 0.394 | -0.018 | 0.118 | 0.883 | 0.019 | 0.102 | 0.850 |  |  |  |  |  |  |  |  |  |  | |  |  |  |
| BMI | 0.003 | 0.004 | 0.459 | 0.012 | 0.005 | **0.017*** | 0.010 | 0.005 | **0.050*** | 0.006 | 0.004 | 0.179 |  |  |  | 0.000 | 0.006 | 0.986 | 0.003 | 0.005 | 0.599 |  | |  |  |  |
| Asthma | 0.165 | 0.142 | 0.253 | 0.321 | 0.152 | **0.041*** | 0.176 | 0.156 | 0.265 | 0.008 | 0.137 | 0.952 |  |  |  | 0.245 | 0.155 | 0.123 |  |  |  |  | |  |  |  |
| Cancer | 0.146 | 0.268 | 0.589 | -0.081 | 0.298 | 0.786 | -0.370 | 0.289 | 0.208 | -0.515 | 0.241 | **0.038*** |  |  |  |  |  |  |  |  |  | -0.605 | | 0.238 | **0.015*** |  |
| COPD | -0.143 | 0.114 | 0.217 | -0.123 | 0.129 | 0.344 | -0.095 | 0.128 | 0.460 | 0.046 | 0.110 | 0.676 |  |  |  |  |  |  |  |  |  |  | |  |  |  |
| CHF | -0.029 | 0.128 | 0.820 | -0.105 | 0.142 | 0.464 | -0.259 | 0.136 | 0.064 | -0.226 | 0.117 | 0.058 |  |  |  |  |  |  | -0.215 | 0.136 | 0.123 | -0.077 | | 0.110 | 0.487 |  |
| T2DM | 0.065 | 0.107 | 0.544 | 0.063 | 0.119 | 0.599 | 0.038 | 0.118 | 0.752 | -0.036 | 0.102 | 0.723 |  |  |  |  |  |  |  |  |  |  | |  |  |  |
| ESRD | 0.195 | 0.163 | 0.237 | -0.227 | 0.179 | 0.211 | -0.279 | 0.175 | 0.118 | -0.471 | 0.139 | **0.002*** |  |  |  |  |  |  |  |  |  | -0.465 | | 0.131 | **0.001*** |  |
| Immunosuppression | 0.014 | 0.151 | 0.925 | -0.228 | 0.167 | 0.178 | -0.067 | 0.167 | 0.692 | -0.081 | 0.142 | 0.571 |  |  |  |  |  |  |  |  |  |  | |  |  |  |
| Max Temperature | 0.043 | 0.064 | 0.501 | 0.092 | 0.071 | 0.199 | 0.071 | 0.070 | 0.318 | 0.025 | 0.061 | 0.685 |  |  |  |  |  |  |  |  |  |  | |  |  |  |
| Days from DSO to max temp | 0.001 | 0.003 | 0.778 | 0.001 | 0.003 | 0.776 | 0.001 | 0.003 | 0.842 | 0.000 | 0.003 | 0.922 |  |  |  |  |  |  |  |  |  |  | |  |  |  |
| Max WBC | 0.001 | 0.004 | 0.822 | -0.001 | 0.004 | 0.834 | -0.003 | 0.004 | 0.543 | -0.003 | 0.004 | 0.378 |  |  |  |  |  |  |  |  |  |  | |  |  |  |
| Days from DSO to max WBC | -0.002 | 0.005 | 0.684 | -0.002 | 0.006 | 0.684 | 0.002 | 0.006 | 0.755 | 0.004 | 0.005 | 0.442 |  |  |  |  |  |  |  |  |  |  | |  |  |  |
| Days from DSO to intubation | -0.005 | 0.008 | 0.535 | -0.017 | 0.008 | **0.044*** | -0.009 | 0.009 | 0.315 | -0.003 | 0.007 | 0.705 |  |  |  | 0.007 | 0.010 | 0.504 |  |  |  |  | |  |  |  |
| P/F Ratio | -0.001 | 0.001 | 0.334 | -0.001 | 0.001 | 0.063 | -0.001 | 0.001 | **0.045*** | -0.001 | 0.000 | 0.195 |  |  |  | -0.001 | 0.001 | 0.213 | -0.001 | 0.001 | 0.265 |  | |  |  |  |
| DVT | -0.052 | 0.142 | 0.717 | -0.115 | 0.158 | 0.471 | 0.042 | 0.157 | 0.790 | 0.086 | 0.134 | 0.526 |  |  |  |  |  |  |  |  |  |  | |  |  |  |
| PE | -0.153 | 0.169 | 0.369 | -0.007 | 0.191 | 0.970 | 0.103 | 0.189 | 0.586 | 0.269 | 0.157 | 0.092 |  |  |  |  |  |  |  |  |  | 0.135 | | 0.140 | 0.340 |  |
| Dexamethasone | 0.268 | 0.374 | 0.478 | -0.176 | 0.416 | 0.674 | -0.300 | 0.409 | 0.467 | -0.568 | 0.344 | 0.106 |  |  |  |  |  |  |  |  |  |  | |  |  |  |
| Tocilizumab | -0.238 | 0.215 | 0.276 | -0.259 | 0.236 | 0.279 | 0.026 | 0.236 | 0.912 | 0.254 | 0.201 | 0.213 |  |  |  |  |  |  |  |  |  |  | |  |  |  |
| Convalescent Plasma | 0.216 | 0.107 | **0.048*** | 0.100 | 0.123 | 0.422 | 0.013 | 0.122 | 0.917 | -0.209 | 0.101 | **0.045*** | 0.215 | 0.105 | **0.046*** |  |  |  |  |  |  | -0.187 | | 0.095 | 0.057 |  |
| Remdesivir | -0.039 | 0.117 | 0.741 | 0.038 | 0.130 | 0.770 | -0.003 | 0.129 | 0.981 | 0.029 | 0.110 | 0.794 |  |  |  |  |  |  |  |  |  |  | |  |  |  |
| VFDs | -0.003 | 0.007 | 0.652 | 0.002 | 0.008 | 0.816 | 0.004 | 0.008 | 0.608 | 0.008 | 0.007 | 0.234 |  |  |  |  |  |  |  |  |  |  | |  |  |  |
| Week 2 x Non-Survivor | -0.390 | 0.194 | **0.049*** | -0.363 | 0.240 | 0.136 | -0.471 | 0.255 | 0.070 | -0.026 | 0.207 | 0.900 | -0.362 | 0.195 | 0.068 | -0.277 | 0.242 | 0.258 | -0.422 | 0.254 | 0.103 | -0.053 | | 0.200 | 0.793 |  |
| Week 3 x Non-Survivor | -0.378 | 0.214 | 0.082 | -0.424 | 0.263 | 0.111 | -0.371 | 0.276 | 0.182 | 0.079 | 0.230 | 0.733 | -0.402 | 0.214 | 0.064 | -0.307 | 0.265 | 0.251 | -0.288 | 0.276 | 0.300 | 0.030 | | 0.220 | 0.892 |  |
| Week 4 x Non-Survivor | -0.438 | 0.249 | 0.082 | -0.104 | 0.302 | 0.731 | -0.387 | 0.315 | 0.222 | 0.093 | 0.268 | 0.730 | -0.447 | 0.247 | 0.075 | -0.025 | 0.302 | 0.934 | -0.336 | 0.313 | 0.287 | 0.113 | | 0.250 | 0.654 |  |
| Week 5 x Non-Survivor | -0.730 | 0.299 | **0.017*** | -0.260 | 0.360 | 0.473 | -0.502 | 0.372 | 0.180 | 0.301 | 0.324 | 0.354 | -0.678 | 0.297 | **0.025*** | -0.160 | 0.362 | 0.659 | -0.488 | 0.370 | 0.191 | 0.378 | | 0.299 | 0.209 |  |
| Week 6 x Non-Survivor | -0.929 | 0.446 | **0.041*** | -1.241 | 0.540 | **0.024*** | -1.304 | 0.558 | **0.022*** | -0.185 | 0.481 | 0.701 | -0.865 | 0.436 | 0.051 | -0.983 | 0.553 | 0.079 | -1.015 | 0.568 | 0.078 | 0.137 | | 0.479 | 0.776 |  |

This table summarizes the estimates from three separate linear mixed-effects model, one for each ceramide species of interest and the C24/C16 ratio. The models included survival status and its interaction with week (relative to DSO) to assess how these factors affected levels of each ceramide species. Unadjusted models were first fitted with demographic and clinical variables, and variables with p-values <0.10 were included in the adjusted models, yielding four final models. Across 53 patients, there were 100 observations for each final model. Bolded numbers with an asterisk represent p-value <0.05.

*Abbreviations used*: BMI, body mass index; C16, ceramide 16:0; C22, ceramide 22:0; C24, ceramide 24:0; CHF, congestive heart failure; COPD, chronic obstructive pulmonary disease; DSO, day of symptom onset; DVT, deep vein thrombosis; ESRD, end-stage renal disease; PE, pulmonary embolism; SE, standard error; T2DM, type 2 diabetes mellitus; VFDs, ventilator-free days; WBC, white blood cell.

**Figure E1. Plasma ceramide ratio levels by survival status.**

**
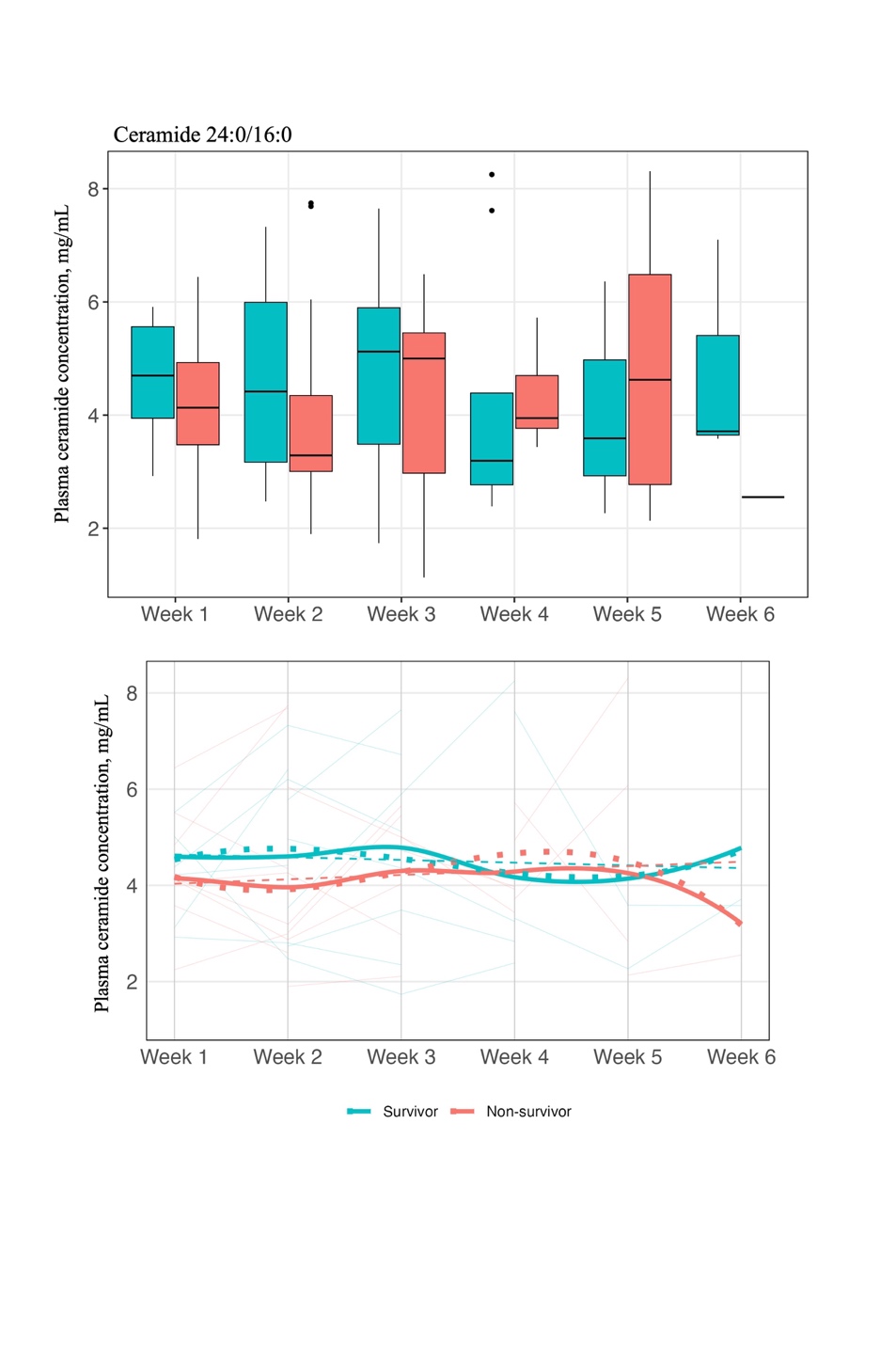
**

This figure depicts C24/C16 levels by survival status. The top presents the data as a box plot, while the bottom shows the temporal trend using line graphs. In the bottom, transparent solid lines indicate individual patient trajectories over time. The dashed line represents the overall linear trend. The dotted line shows a flexible spline-based trend using a cubic B-spline regression, and the solid bold line depicts a smoothed LOESS curve summarizing the average trend. Data presented in red represent non-survivors, while those presented in teal represent survivors.

*Abbreviations used*: LOESS, locally estimated scatterplot smoothing.
